# Misconceptions About Traumatic Brain Injuries in Five Sub-Saharan African Countries

**DOI:** 10.7759/cureus.18369

**Published:** 2021-09-29

**Authors:** Oloruntoba Ogunfolaji, Chinedu Egu, Lorraine Sebopelo, Dawin Sichimba, Yvan Zolo, Crecencia Mashauri, Emmanuel Phiri, Neontle Sakaiwa, Andrew Alalade, Ulrick Sidney Kanmounye

**Affiliations:** 1 General Medicine, College of Medicine, University of Ibadan, Ibadan, NGA; 2 Department of Research, Association of Future African Neurosurgeons, Yaounde, CMR; 3 Neurological Surgery, Queens Medical Centre, Nottingham University Hospitals, Nottingham, GBR; 4 Faculty of Medicine, University of Botswana, Gaborone, BWA; 5 Faculty of Medicine, Michael Chilufya Sata School of Medicine, Copperbelt University, Kitwe, ZMB; 6 Faculty of Health Sciences, University of Buea, Buea, CMR; 7 Faculty of Medicine, Medical Institute, Derzhavin Tambov State University, Tambov, RUS; 8 Neurosurgery, Royal Preston Hospital, Preston, GBR; 9 Neurosurgery, Bel Campus University of Technology, Kinshasa, COD; 10 Global Neurosurgery Initiative, Harvard Medical School, Boston, USA

**Keywords:** traumatic brain injury, sub-saharan africa, misconceptions, brain injury, africa

## Abstract

Background

Traumatic Brain Injury (TBI) remains a significant problem in certain regions of the world but receives little attention despite its enormous burden. This discrepancy could consequently lead to various misconceptions among the general public. This study evaluated misconceptions about TBI in five African countries.

Methods

Data for this cross-sectional study were collected using the Common Misconception about Traumatic Brain Injury (CM-TBI) questionnaire, which was electronically disseminated from January 16 to February 6, 2021. Associations between the percentage of correct answers and independent variables (i.e., sociodemographic characteristics and experience with TBI) were evaluated with the ANOVA test. Additionally, answers to the question items were compared against independent variables using the Chi-Square test. A P-value <0.05 was considered statistically significant.

Results

A total of 817 adults, 50.2% female (n=410), aged 24.3 ± 4.3 years, and majoritarily urban dwellers (94.6%, n=773) responded to the survey. They had received tertiary education (79.2%, n=647) and were from Nigeria (77.7%, n=635). Respondents had few misconceptions (mean correct answers=71.7%, 95% CI=71.0-72.4%) and the amnesia domain had the highest level of misconception (39.3%, 95% CI=37.7-40.8%). Surveyees whose friends had TBI were more knowledgeable about TBI (mean score difference=4.1%, 95% CI=1.2-6.9, P=0.01). Additionally, surveyees whose family members had experienced TBI had a better understanding of brain damage (mean score difference=5.7%, 95% CI=2.1-9.2%, P=0.002) and recovery (mean score difference=4.3%, 95% CI=0.40-8.2%, P=0.03).

Conclusion

This study identified some misconceptions about TBI among young adult Africans. This at-risk population should benefit from targeted education strategies to prevent TBI and reduce TBI patients' stigmatization in Africa.

## Introduction

According to the World Health Organisation (WHO), Traumatic brain injury (TBI) is a major cause of death and disability [1]. TBI contributes significantly to morbidity and mortality in low- and middle-income countries (LMICs), especially in sub-Saharan Africa (SSA) [2].

In 2019, Africa's total population was estimated at 1.3 billion, about 16.8% of the world's population. Africa's population pyramid is expansive; 67.4% of its population is less than 30 years, 21.3% is between 30-49, and 11.3% is aged 50 or more [3]. The continent's population is likely to reach 2.49 billion by 2050 (about 26% of the world's total) and 4.28 billion by 2100 (about 39% of the world's total) according to the United Nations' estimates [3]. 

Of the 27,082,033 incidents of TBI cases recorded globally in 2016, 10.9% (i.e, 2,956,908 cases) were recorded in SSA [4]. There has also been an increasing trend in the number of prevalent and incident TBI cases within the region over the past 15 years. The huge and increasing incidence of TBI in SSA has been attributed to increased transportation (car, motorcycle, and bicycle) use and inobservance of road traffic laws [5,6].

Despite the large and increasing burden of TBI in SSA, TBI gets little attention. The lack of attention has fostered the development of misconceptions in the general public. These misconceptions translate into an increase in the burden of TBI and the stigmatization of TBI patients. In a study of discharged TBI patients, Pappadis et al. observed that 76-83% of respondents held erroneous beliefs about post-TBI amnesia and recovery [7]. In a similar study, Guilmette and Paglia found that 60% of discharged TBI patients believed that severe TBI does not affect vocational functioning [8].

In contrast, two-thirds believed that an X-ray was the only way to investigate TBI. A study of college students by Ernst et al. [9] found all students held misconceptions about TBI; however, non-nursing students were more likely to hold misconceptions than nursing students. Interestingly, these misconceptions existed among nursing students despite their clinical exposure to TBI patients.

No study has evaluated misconceptions among adults from multiple SSA countries. This study aims to analyze the misconceptions about TBI among young adult Africans and to evaluate any associations of any TBI-related misconceptions with the various sociodemographic attributes.

## Materials and methods

Data collection tool

The authors used a three-part questionnaire. The first part had six questions on sociodemographic characteristics. The second part evaluated the TBI experience (four questions). The last part was a standardized instrument - the Common Misconceptions about Traumatic Brain Injury (CM-TBI) questionnaire. The CM-TBI is a 40-item self-report questionnaire that explores misconceptions related to TBI Prevention, Brain damage, Brain injury sequelae, Unconsciousness, Amnesia, Recovery process, and Rehabilitation. Respondents answered True or False to question prompts on the CM-TBI. This questionnaire has been validated and used by numerous authors [10,11]. 

Data collection

The e-survey was hosted on Google Forms (Google, CA, USA) and distributed via WhatsApp (Facebook Inc., CA, USA) to adults in Botswana, Cameroon, Nigeria, Tanzania, and Uganda from January 16, to February 6, 2021, as the authors were from these countries and the countries represented the major sub-regions in sub-Saharan Africa. A convenient sampling method was used, and respondent anonymity was preserved. Participation in the study was voluntary, and no financial incentivization was involved. Respondents consented and were free to drop out or decline to answer a question whenever they chose. The data were stored in a password-protected account, and access to the data was limited. The survey data can be accessed on Open Science Framework (https://osf.io/xkf34/). A waiver was obtained from the institutional review board of the Bel Campus University of Technology.

Data analysis

Age was expressed as a mean and standard deviation; all categorical variables were reported as frequencies and percentages. The association between categorical independent factors (i.e., sociodemographic characteristics and TBI experience) and the number of correct item responses were evaluated with the ANOVA test. Correct answers were scored 1 and incorrect answers were scored 0 so that higher scores meant fewer misconceptions. Also, the association between age and correct responses was evaluated using Pearson's correlation. Each assertion was compared against sociodemographic characteristics and TBI experience using the Chi-Square test. A P-value<0.05 was considered statistically significant. The data were analyzed with SPSS statistics v. 26 (IBM corp., Armonk, NY).

## Results

The survey was answered by 817 people from: Nigeria (77.7%, n=635), Tanzania (8.0%, n=65), Zambia (5.9%, 48), Cameroon (4.4%, n=36), and Botswana (4.0%, n=33). The respondents were 24.3 ± 4.3 years old, 410 (50.2%) were female, 773 (94.6%) were urban dwellers, and 647 (79.2%) had received or were receiving tertiary education. Only 19 (2.3%) respondents had experienced a TBI. However, 100 (12.2%) had a family who had experienced TBI. Two hundred and fifty (30.6%) respondents had witnessed TBI, the most common cause of experienced/witnessed TBI was motor vehicle accidents (31.7%, n=259) followed by bike accidents (16.3%, n=133) (Table 1).

**Table 1:** Sociodemographic characteristics and traumatic brain injury experience of survey respondents

Characteristic	Frequency (Percentage)
Sex	
Female	410 (50.2)
Male	407 (49.8)
Country	
Botswana	33 (4.0)
Cameroon	36 (4.4)
Nigeria	635 (77.7)
Tanzania	65 (8.0)
Zambia	48 (5.9)
Place of Residence	
Rural	44 (5.4)
Urban	773 (94.6)
Highest Level of Education	
Secondary	170 (20.8)
Tertiary	647 (79.2)
Have you or someone you know experienced traumatic brain injury?	
Myself	19 (2.3)
Family member	100 (12.2)
Friend	197 (24.1)
Distant acquaintance	15 (1.8)
No one I know of, myself included	117 (14.3)
Witnessed a traumatic brain injury	250 (30.6)
Cause of witnessed/experienced traumatic brain injury	
Motor vehicle accident	259 (31.7)
Bike accident	133 (16.3)
Domestic or work accident	128 (15.7)
Assault	91 (11.1)
Pedestrian vehicle accident	80 (9.8)

Respondents had few misconceptions (mean correct answers=71.7%, 95% CI=71.0-72.4%). Most surveyees responded correctly to prevention (80.9%, 95% CI=79.7-82.2%), brain damage (88.7%, 95% CI=87.6-89.9%), brain injury (82.1%, 95% CI=81.0-83.1%), unconsciousness (67.5%, 95% CI=65.5-69.6%), recovery (67.4%, 95% CI=66.1-68.7%), and rehabilitation (75.8%, 95% CI=74.1-77.6%).

Amnesia had the lowest correct responses (39.3%, 95% CI=37.7-40.8%). Most respondents believed a person with a brain injury may have trouble remembering events that happened before the injury, but usually does not have trouble learning new things (76.4%, n=624) and that people with a brain injury can forget who they are and not recognize others, but be normal in every other way (91.6%, n=748).

Respondents were almost evenly split on the following question prompts (Table 2):

It is safer to be trapped inside a wreck than to be thrown clear: False (44.9%, n=367) vs. True (55.1%, n=450);

It is common for people with brain injuries to be easily angered: False (50.3%, n=411) vs. True (49.7%, n=406);

Asking persons who have had a brain injury about their progress is the most accurate, informative way to find out how they have progressed: False (48.2%, n=394) vs. True (51.8%, n=423);

How quickly a person recovers depends mainly on how hard he or she works at recovering: False (50.4%, n=412) vs. True (49.6%, n=405);

The primary goal of brain injury rehabilitation is to increase physical abilities such as walking: False (52.3%, n=427) vs. True (47.7%, n=390).

**Table 2:** Conceptions about traumatic brain injury

Questions [Correct response: False (F), True (T)]	False [N, (%)]	True [N, (%)]
Prevention		
You don't need seatbelts as long as you can brace yourself before a crash (F)	808 (98.9)	9 (1.1)
It is more important to use seatbelts on long trips than in driving around town (F)	640 (78.3)	177 (20.7)
It is safer to be trapped inside a wreck than to be thrown clear (T)	367 (44.9)	450 (55.1)
Wearing seatbelts causes as many injuries as it prevents (F)	747 (91.4)	70 (8.6)
Brain Damage		
A head injury can cause brain damage even if the person is not knocked out (T)	57 (7.0)	760 (93.0)
A little brain damage doesn't matter much, since people only use a part of their brains anyway (F)	796 (97.4)	21 (2.6)
It is obvious that someone has brain damage because they look different from people who don't have brain damage (F)	713 (87.3)	104 (12.7)
Whiplash injuries to the neck can cause brain damage even if there is no direct blow to the head (T)	186 (22.8)	631 (77.2)
Brain injury sequelae		
It is common for people with brain injuries to be easily angered (T)	411 (50.3)	406 (49.7)
It is possible that a person's personality will change after a brain injury (T)	68 (8.3)	749 (91.7)
Problems with speech, coordination, and walking can be caused by brain damage (T)	28 (3.4)	789 (96.6)
Problems with irritability and difficulties controlling anger are common in people who have had a brain injury (T)	202 (24.7)	615 (75.3)
Most people with brain damage are not fully aware of its effect on their behavior (T)	96 (11.8)	721 (88.2)
Brain injury patients usually show a good understanding of their problems because they experience them every day (F)	603 (73.8)	214 (26.2)
Brain injuries may cause one to feel depressed, sad, and hopeless (T)	43 (5.3)	774 (94.7)
Drinking alcohol may affect a person differently after a brain injury (T)	183 (22.4)	634 (77.6)
It is common for people to experience changes in behavior after a brain injury (T)	72 (8.8)	745 (91.2)
Unconsciousness		
When people are knocked unconscious, most wake up quickly with no lasting effects (F)	466 (57.0)	351 (43.0)
People in a coma are usually not aware of what is happening around them (T)	170 (20.8)	647 (79.2)
Even after several weeks in a coma, when people wake up, most recognize and speak to others right away (F)	542 (66.3)	275 (33.7)
Amnesia		
People usually have more trouble remembering things that happen after an injury than remembering things from before (T)	333 (40.8)	484 (59.2)
Sometimes a second blow to the head can help a person remember things that were forgotten (F)	537 (65.7)	280 (34.3)
A person with a brain injury may have trouble remembering events that happened before the injury, but usually does not have trouble learning new things (F)	193 (23.6)	624 (76.4)
People with a brain injury can forget who they are and not recognize others, but be normal in every other way (F)	69 (8.4)	748 (91.6)
Recovery		
Recovery from a brain injury usually is complete in about 5 months (F)	630 (77.1)	187 (22.9)
Complete recovery from a severe brain injury is not possible, no matter how badly the person wants to recover (T)	495 (60.6)	322 (39.4)
Once a person is able to walk again, his/her brain is almost fully recovered (F)	643 (78.7)	174 (21.3)
Slow recovery may continue even 1 year after injury (T)	46 (5.6)	771 (94.4)
It is necessary for a person to go through a lot of physical pain to recover from a brain injury (F)	580 (71.0)	237 (29.0)
Once a person with a brain injury realizes where they are, they will always be aware of this (F)	459 (56.2)	358 (43.8)
A person who has recovered from a head injury is less able to withstand a second blow to the head (T)	271 (33.2)	546 (66.8)
Asking persons who have had a brain injury about their progress is the most accurate, informative way to find out how they have progressed (F)	394 (48.2)	423 (51.8)
It is good advice to remain completely inactive during recovery from a brain injury (F)	667 (81.6)	150 (18.4)
Once a person recovering from a brain injury feels 'back to normal' the recovery process is complete (F)	633 (77.5)	184 (22.5)
How quickly a person recovers depends mainly on how hard he or she works at recovering (F)	412 (50.4)	405 (49.6)
Rehabilitation		
'Cognitive' refers to thinking processes such as memory, attention, and learning (T)	26 (3.2)	791 (96.8)
'Cognitive' refers to the ability to move your body (F)	641 (78.5)	176 (21.5)
The primary goal of brain injury rehabilitation is to increase physical abilities such as walking (F)	427 (52.3)	390 (47.7)

Experience with TBI

Respondents whose family members had experienced TBI had higher scores in the brain damage (mean score difference=5.7%, 95% CI=2.1-9.2%, P=0.002) and recovery (mean score difference=4.3%, 95% CI=0.40-8.2%, P=0.03) sections. Additionally, they were less likely to reject the claim that it is obvious that someone has brain damage because they look different from people who don't have brain damage (odds ratio (OR)=0.40, 95% CI=0.17-0.95, P=0.03) or that once a person is able to walk again, his/her brain is almost fully recovered (OR=0.29, 95% CI=0.14-0.61, P=0.001). Similarly, they were less likely to disagree with the claims it is necessary for a person to go through a lot of physical pain to recover from a brain injury (OR=0.50, 95% CI=0.29-0.85, P=0.01) and that 'cognitive' refers to the ability to move your body (OR=0.51, 95% CI=0.28-0.93, P=0.03). However, they did not believe whiplash injuries to the neck can cause brain damage even if there is no direct blow to the head (OR=2.35, 95% CI=1.26-4.40, P=0.01).

Respondents whose friends had a TBI scored higher than other participants (mean score difference=4.1%, 95% CI=1.2-6.9, P=0.01). In addition, they were less likely to reject the following statements: it is more important to use seatbelts on long trips than in driving around town (OR=0.58, 95% CI=0.38-0.89, P=0.01); and even after several weeks in a coma, when people wake up, most recognise and speak to others right away (OR=0.58, 95% CI=0.40-0.83, P=0.003). They did not believe it is obvious that someone has brain damage because they look different from people who don't have brain damage (OR=1.73, 95% CI=1.11-2.67, P=0.02) or that complete recovery from a severe brain injury is not possible, no matter how badly the person wants to recover (OR=1.41, 95% CI=1.02-1.95, P=0.04). Also, they disagreed with the claim that once a person is able to walk again, his/her brain is almost fully recovered (OR=1.57, 95% CI=1.09-2.28, P=0.02) and that the primary goal of brain injury rehabilitation is to increase physical abilities such as walking (OR=1.58, 95% CI=1.14-2.18, P=0.01).

Surveyees whose distant acquaintances had experienced TBI did not believe that when people are knocked unconscious, most wake up quickly with no lasting effects (OR=3.74, 95% CI=1.18-11.84, P=0.02) and that complete recovery from a severe brain injury is not possible, no matter how badly the person wants to recover (OR=3.14, 95% CI=1.06-9.28, P=0.03). In contrast, they were less likely to oppose the claim that even after several weeks in a coma, when people wake up, most recognize and speak to others right away (OR=0.14, 95% CI=0.02-1.05, P=0.03).

Respondents who had not experienced TBI and knew no one who had experienced TBI did not believe that drinking alcohol may affect a person differently after a brain injury (OR=1.83, 95% CI=1.06-3.15, P=0.03).

Respondents who had witnessed a TBI had higher total scores (mean score difference=1.6%, 95% CI=0.1-3.1%, P=0.03): especially in the brain damage (mean score difference=2.6%, 95% CI=0.05-5.1%, P=0.04) and amnesia (mean score difference=3.6%, 95% CI=0.3-6.9%, P=0.03) sections. These respondents did not believe that whiplash injuries to the neck can cause brain damage even if there is no direct blow to the head (OR=1.91, 95% CI=1.29-2.82, P=0.001). However, they were more likely to oppose the assertion that most people with brain damage are not fully aware of its effect on their behavior (OR=0.60, 95% CI=0.39-0.93, P=0.02) and that even after several weeks in a coma, when people wake up, most recognise and speak to others right away (OR=0.70, 95% CI=0.51-0.97, P=0.03). They were more likely to disagree with the fact that a person with a brain injury may have trouble remembering events that happened before the injury, but usually does not have trouble learning new things (OR=0.69, 95% CI=0.49-0.97, P=0.03).

Education

Respondents who had received tertiary education had lower scores on the recovery section than those who had received secondary education only [mean score difference=-3.3%, 95% CI=-6.4-(-0.1)%, P=0.04]. Survey participants with university-level education did not believe that it is common for people with brain injuries to be easily angered (OR=1.59, 95% CI=1.13-2.24, P=0.01) or that most people with brain damage are not fully aware of its effect on their behavior (OR=1.78, 95% CI=1.11-2.86, P=0.02). Additionally, they did not believe that asking persons who have had a brain injury about their progress is the most accurate, informative way to find out how they have progressed (OR=1.52, 95% CI=1.08-2.14, P=0.02) or that it is good advice to remain completely inactive during recovery from a brain injury (OR=1.66, 95% CI=1.02-2.70, P=0.04).

Sex

More female surveyees did not believe that how quickly a person recovers depends mainly on how hard he or she works at recovering (OR=1.38, 95% CI=1.05-1.81, P=0.02).

Age

There was no significant correlation between age and correct responses (Table 3).

**Table 3:** Correlation between age and the proportion of correct responses

Question topic	Pearson correlation	P-value
Prevention	0.02	0.61
Brain damage	0.05	0.13
Brain injury	0.03	0.40
Unconsciousness	0.01	0.88
Amnesia	0.02	0.49
Recovery	0.02	0.62
Rehabilitation	0.01	0.75
Total	0,04	0.22

## Discussion

Key findings

This study is the first to evaluate the misconceptions about TBI from all SSA regions (Central, Eastern, Southern, and Western Africa). Misconceptions were significantly associated with having experienced TBI or having an acquaintance who had experienced TBI. The largest proportion of misconceptions concerned post-TBI amnesia, and this misconception was less common among people who had witnessed a TBI.

Pappadis et al. found an association between misconceptions and the level of education, religion, place of birth, and race [7]. However, Gurusamy et al. did not find a statistically significant association between misconceptions about TBI and sex, level of education, place of residence, and prior exposure to TBI among Indian nursing students [10]. Gurusamy et al. aggregated misconception data and then compared the total misconception score with sociodemographic characteristics and TBI exposure [10]. Our study analyzed the relationships between independent variables, and i. aggregated (question blocks) and ii. disaggregated (question items) data. Our findings show that certain groups demonstrated variable degrees of misconceptions. For example, respondents whose distant acquaintance(s) had experienced TBI were up to 11 times more likely to reject the fact that most wake up quickly with no lasting effects when people are knocked unconscious. However, these respondents were up to nine times more likely to incorrectly reject the assertion that complete recovery from a severe brain injury is not possible, no matter how badly the person wants to recover. These differences highlight the necessity to target TBI education campaigns in certain groups. The distribution of Young vs. Old and Rural vs. Urban dwellers were similar in the target countries and as such, misconception levels between these countries were positively correlated and this is similar to findings by Gurusamy et al. [10].

Amnesia

Like in our study, Pretorius and Broodryk found that the greatest rates of misconceptions were about post-TBI amnesia [11]. SSA adults believed a person with a brain injury may have trouble remembering events that happened before the injury, but usually does not have trouble learning new things and people with a brain injury can forget who they are and not recognize others, but be normal in every other way. Amnesia is a loss of memory, and when it is secondary to trauma, it is called posttraumatic amnesia. Cantu et al. classified posttraumatic amnesia into two: i. anterograde amnesia, i.e., the inability to retain new knowledge after the incident, and ii. retrograde amnesia, i.e., the inability to recall events that occurred immediately before the incident [12]. Patients can experience both retrograde and anterograde amnesia, and this depends on the severity of TBI [13]. Of note, post-TBI amnesia duration is not linked with intellectual disability [14], and repeated blows to the head may cause diffuse axonal injury, worsen amnesia, or result in other complications such as loss of consciousness [15]. This finding has dispelled the popularly held belief that second blows to the head may lead to memory recollection [15].

Prevention

Most respondents in our study had a good understanding of road safety measures like the use of seatbelts, the rationale behind these measures, and the negative consequences that might arise when road traffic laws are not respected. Road transport is the main mode of transportation in SSA [16]. Paradoxically, SSA countries have poor road systems, which contribute to the burden of road traffic accidents [17]. The non-adherence of road traffic laws further compounds the burden of road traffic accidents in SSA [18]. SSA governments and stakeholders have invested in road transportation infrastructure and developed campaigns to increase the dissemination and adherence to road traffic rules [19]. The campaigns have focused on urban dwellers, pedestrians, motorcycle users, and schools [19]. The campaigns are run on TV, radio, and social media to better reach the target audience [20]. These public health considerations might explain why our respondents had a good understanding of TBI prevention.

Despite road traffic adherence campaigns' success, road traffic accidents remain the first cause of TBI in SSA, and the level of adherence to road traffic laws remains low [5]. This suggests that awareness of road traffic laws is not translating into behavioral changes among SSA road users. More research is needed to understand the impact of road traffic law education and advocacy on behavior changes.

Brain damage

Most respondents believed that it is obvious that someone has brain damage because they look different from people who don't have brain damage. A TBI can result in both implicit and explicit deficits. Implicit deficits are less observable and include psychosocial, behavioral, and cognitive problems [8]. These include disorders of memory, attention, concentration, mood, and personality. Explicit deficits are more observable and may include motor, sensory, and speech problems [8]. 

Whiplash injuries to the neck can indeed cause brain damage even if there is no direct blow to the head. The transmission of impact forces characterizes whiplash injuries to the cervical spine leading to stress impacting the brain, ultimately causing brain injury [21]. Memory and concentration impairment are the ultimate sequelae of these injuries [22].

Coma

In our study, most respondents correctly answered questions on posttraumatic coma. Misconceptions about the sequelae of coma were associated with TBI experience, i.e., having witnessed TBI, having a friend who has had TBI, and having a distant acquaintance who had a TBI. Similarly, misconceptions about TBI duration were associated with having a distant acquaintance who had a TBI.

Coma is usually a transitory state though it may last for an indefinite or even prolonged period [23]. Alerting and arousal functions of the brain are affected and awareness and the content of consciousness, which translates to unresponsiveness to the environment. Studies suggest that age, Glasgow Coma Scale (GCS), pupil reaction and eye movements, CT-scan findings, duration of posttraumatic amnesia, brainstem dysfunction, and intracranial pressure (ICP) or a combination of all these factors affect post-TBI coma outcomes [24-26]. GCS during the first 24 hours post-TBI is predictive of coma outcome in the first two to three days after the injury [25]. Also, age < 40 years is associated with rapid recovery within the first days and weeks post-TBI [24]. A more specific positive prognostic factor is an electroencephalogram (EEG) showing reactivity to external stimulation [24].

Rehabilitation

Some respondents incorrectly believed that the primary aim of rehabilitation is to increase physical ability such as walking. Rehabilitation is a holistic treatment and aims to improve "the overall feeling of well-being, quality, and satisfaction of life of TBI patients and their families while respecting their free will." [27]. 

Limitations

We acknowledge the following limitations to our study. There was low representation from countries other than Nigeria. Nigeria's population is greater than that of Botswana, Cameroon, Tanzania, and Zambia combined. We believe our sample population reflected this. In this study, people with low education levels or residents in rural areas constituted a small percentage. Despite their underrepresentation, we found a statistically significant association between the level of education and misconceptions about TBI. However, we did not find statistically significant associations between misconceptions about TBI and location (rural and urban).

## Conclusions

TBI affects millions of people each year, especially young adults - disabling many of them for life. The funding allocated to and research on TBI is not proportional to its burden. Advocacy and education are critical to increasing TBI awareness among the general public and stakeholders. Our study has identified misconceptions among young adult Africans - the at-risk population for TBI - and other sociodemographic characteristics associated with these misconceptions. These findings can help develop targeted education strategies aimed at preventing TBI, advocating for increased funding and research, and reducing TBI patients' stigmatization in Africa. Awareness of these findings is important for global neurosurgery specialists, TBI patients and their families, policymakers, and the general public. 
